# *N*′-[(*E*)-2-Meth­oxy­benzyl­idene]pyrazine-2-carbohydrazide

**DOI:** 10.1107/S1600536811022938

**Published:** 2011-06-18

**Authors:** Marcus V. N. de Souza, Camilo H. da Silva Lima, James L. Wardell, Solange M. S. V. Wardell, Edward R. T. Tiekink

**Affiliations:** a Fundação Oswaldo Cruz, Instituto de Tecnologia em Fármacos – Farmanguinhos, R. Sizenando Nabuco, 100, Manguinhos, 21041-250 Rio de Janeiro, RJ, Brazil; b Centro de Desenvolvimento Tecnológico em Saúde (CDTS), Fundação Oswaldo Cruz (FIOCRUZ), Casa Amarela, Campus de Manguinhos, Av. Brasil 4365, 21040-900 Rio de Janeiro, RJ, Brazil; c CHEMSOL, 1 Harcourt Road, Aberdeen AB15 5NY, Scotland; d Department of Chemistry, University of Malaya, 50603 Kuala Lumpur, Malaysia

## Abstract

In the title compound, C_13_H_12_N_4_O_2_, all the non-H atoms lie on a crystallographic mirror plane and an intra­molecular N—H⋯N hydrogen bond generates an *S*(5) ring; the conformation about the imine bond [1.280 (3) Å] is *E*. In the crystal, mol­ecules assemble into a two-dimensional array *via* C—H⋯O(carbon­yl) and C—H⋯N(pyrazine) contacts. Layers stack along the *b*-axis direction *via* weak π–π inter­actions between pyrazine rings [ring centroid distance = 3.8028 (8) Å].

## Related literature

For background to the anti-mycobacterial activity of pyrazin­amide derivatives, see: Chaisson *et al.* (2002 ▶); Gordin *et al.* (2000 ▶); de Souza (2006 ▶); Pinheiro *et al.* (2007 ▶). For related structures of pyrazine­carbonyl­hydrazones, see: Baddeley *et al.* (2009 ▶); Howie *et al.* (2010*a* ▶,*b* ▶).
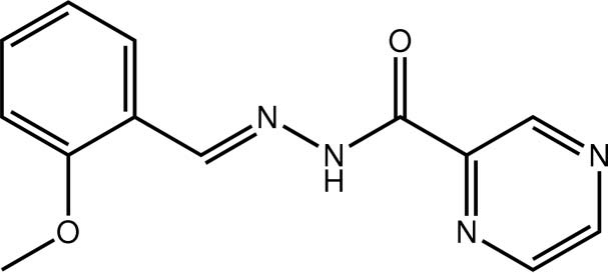


## Experimental

### 

#### Crystal data

C_13_H_12_N_4_O_2_
*M* *_r_* = 256.27
Monoclinic, 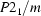

*a* = 7.7615 (6) Å
*b* = 6.4257 (4) Å
*c* = 12.2480 (9) Å
β = 93.893 (3)°
*V* = 609.44 (8) Å^3^
*Z* = 2
Mo *K*α radiation
μ = 0.10 mm^−1^
*T* = 120 K
0.46 × 0.24 × 0.01 mm

#### Data collection

Nonius KappaCCD area-detector diffractometer
Absorption correction: multi-scan (*SADABS*; Sheldrick, 2007 ▶) *T*_min_ = 0.594, *T*_max_ = 0.746
7850 measured reflections
1481 independent reflections
1032 reflections with *I* > 2σ(*I*)
*R*_int_ = 0.074

#### Refinement

*R*[*F* ^2^ > 2σ(*F* ^2^)] = 0.052
*wR*(*F* ^2^) = 0.147
*S* = 1.03
1481 reflections
122 parameters
4 restraints
H atoms treated by a mixture of independent and constrained refinement
Δρ_max_ = 0.31 e Å^−3^
Δρ_min_ = −0.29 e Å^−3^

### 

Data collection: *COLLECT* (Hooft, 1998 ▶); cell refinement: *DENZO* (Otwinowski & Minor, 1997 ▶) and *COLLECT*; data reduction: *DENZO* and *COLLECT*; program(s) used to solve structure: *SHELXS97* (Sheldrick, 2008 ▶); program(s) used to refine structure: *SHELXL97* (Sheldrick, 2008 ▶); molecular graphics: *ORTEP-3* (Farrugia, 1997 ▶), *DIAMOND* (Brandenburg, 2006 ▶); software used to prepare material for publication: *PLATON* (Spek, 2009 ▶) and *publCIF* (Westrip, 2010 ▶).

## Supplementary Material

Crystal structure: contains datablock(s) global, I. DOI: 10.1107/S1600536811022938/hb5910sup1.cif

Structure factors: contains datablock(s) I. DOI: 10.1107/S1600536811022938/hb5910Isup2.hkl

Additional supplementary materials:  crystallographic information; 3D view; checkCIF report

## Figures and Tables

**Table 1:** Hydrogen-bond geometry (Å, °)

*D*—H⋯*A*	*D*—H	H⋯*A*	*D*⋯*A*	*D*—H⋯*A*
N3—H3n⋯N1	0.88 (2)	2.27 (2)	2.685 (3)	109 (2)
C2—H2⋯O1^i^	0.95	2.57	3.160 (3)	121
C3—H3⋯O1^i^	0.95	2.44	3.103 (3)	127
C9—H9⋯N2^ii^	0.95	2.56	3.437 (3)	153

Symmetry codes: (i) 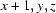; (ii) 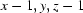.

**Fig. 1.**
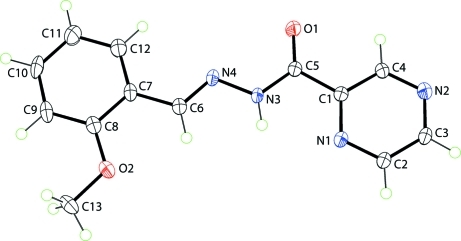
The molecular structure of (I) showing displacement ellipsoids at the 50% probability level.

**Fig. 2.**
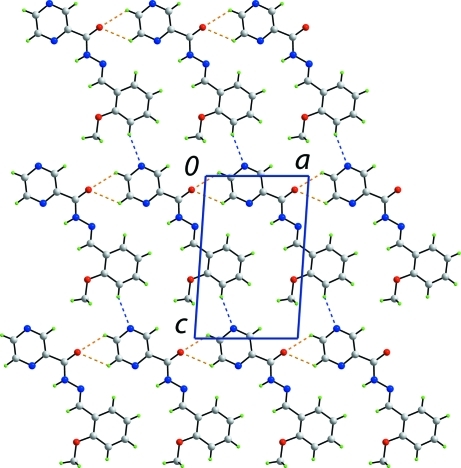
View of the supramolecular array in the ac plane in the crystal structure of (I). The C—H···O and C—H···N contacts are shown as orange and blue dashed lines, respectively.

**Fig. 3.**
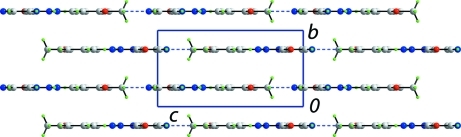
A view in projection down the b axis of the unit-cell contents of (I) with the C—H···N contacts shown as blue dashed lines.

## supplementary crystallographic information

### Comment

Pyrazinamide has well known anti-mycobacterial activity and it is the one of the most important drugs used in tuberculosis treatment (Chaisson *et al.*, 2002; Gordin *et al.*, 2000; de Souza, 2006). Various derivatives have been prepared and their anti-tuberculosis properties studied (Pinheiro *et al.*, 2007). Among the reported crystal structures of pyrazinamide derivatives are those of pyrazinecarbonylhydrazones (Baddeley *et al.*, 2009; Howie *et al.* 2010*a*, 2010*b*). In continuation of previous work, we now wish to report on the crystal structure of the title compound, (I).

All non-hydrogen atoms in (I) lie on a crystallographic mirror plane, Fig. 1. The formation of intramolecular N3—H···N1 and C6—H···O2 contacts, Table 1, contributes to the stability of the planar conformation. The configuration about the N4═C6 bond [1.280 (3) Å] is *E*. The most prominent contacts in the crystal packing are of the type C—H···O and C—H···N, Table 1. The C—H···O contacts lead to chains along the *a* direction and involve the bifurcated carbonyl-O1 atom. Chains are linked in the *c* direction by C—H···N2(pyrazinyl) contacts with the result that a two-dimensional array is formed in the *ac* plane, Fig. 2. Layers stack along the *b* direction stabilized by weak π–π interactions formed between pyrazinyl rings [ring centroid(N1,N2,C1–C4)···ring centroid(N1,N2,C1—C4)^i^ = 3.8028 (8) Å for *i*: 1 - *x*, -1/2 + *y*, 2 - *z*], Fig. 3.

### Experimental

Solutions of 2-pyrazinehydrazide (0.72 mmol) in water (10 ml), and 2-methoxybenzaldehyde (0.79 mmol) in ethanol (10 ml) were mixed and the reaction mixture was stirred at ambient temperature, until TLC indicated reaction was complete. The solvent was removed under reduced pressure and the residue was washed with cold diethyl ether (30 ml) and recrystallized from ethanol to yield colourless plates of (I). Yield: 52%; *M*.pt.: 453–454 K. ^1^H NMR (400 MHz, DMSO-d6) δ: 12.35 (1*H*, s, NH), 9.26 (1*H*, s, H3), 9.00 (1*H*, s, H6), 8.92 (1*H*, s, H5), 8.80 (1*H*, s, N═CH), 7.91 (1*H*, d, J=7.5 Hz, H60), 7.45 (1*H*, t, J=7.5, H40), 7.12 (1*H*, d, J=7.5 Hz, H3'), 7.04 (1*H*, t, J=7.5 Hz, H5'), 3.87 (3*H*, s, OCH_3_) p.p.m.. ^13^C NMR (100 MHz, DMSO-d6) δ: 160.5, 148.8, 148.2, 144.7, 144.4, 143.7, 133.5, 133.2, 132.2, 128.4, 127.8, 124.1, 55.4 p.p.m. MS/ESI: [M—H]: 255. IR (KBr pellets): \v 3300 (N—H); 1680 (C═O) cm^-1^.

### Refinement

The C-bound H atoms were geometrically placed (C—H = 0.95–0.98 Å) and refined as riding with *U*_iso_(H) = 1.2–1.5*U*_eq_(C). The N-bound atom was refined with N—H = 0.88±0.01 Å, and with *U*_iso_(H) = 1.2*U*_eq_(N). One reflection, *i.e*. (011), was omitted from the final refinement owing to poor agreement.

### Crystal data

**Table:**

C_13_H_12_N_4_O_2_	*F*(000) = 268
*M**_r_* = 256.27	*D*_x_ = 1.397 Mg m^−^^3^
Monoclinic, *P*2_1_/*m*	Mo *K*α radiation, λ = 0.71073 Å
Hall symbol: -P 2yb	Cell parameters from 6063 reflections
*a* = 7.7615 (6) Å	θ = 2.9–27.5°
*b* = 6.4257 (4) Å	µ = 0.10 mm^−^^1^
*c* = 12.2480 (9) Å	*T* = 120 K
β = 93.893 (3)°	Plate, colourless
*V* = 609.44 (8) Å^3^	0.46 × 0.24 × 0.01 mm
*Z* = 2	

### Data collection

**Table:**

Nonius KappaCCD area-detector diffractometer	1481 independent reflections
Radiation source: Enraf Nonius FR591 rotating anode	1032 reflections with *I* > 2σ(*I*)
10 cm confocal mirrors	*R*_int_ = 0.074
Detector resolution: 9.091 pixels mm^-1^	θ_max_ = 27.4°, θ_min_ = 3.0°
φ and ω scans	*h* = −9→9
Absorption correction: multi-scan (*SADABS*; Sheldrick, 2007)	*k* = −8→8
*T*_min_ = 0.594, *T*_max_ = 0.746	*l* = −14→15
7850 measured reflections	

### Refinement

**Table:**

Refinement on *F*^2^	Primary atom site location: structure-invariant direct methods
Least-squares matrix: full	Secondary atom site location: difference Fourier map
*R*[*F*^2^ > 2σ(*F*^2^)] = 0.052	Hydrogen site location: inferred from neighbouring sites
*wR*(*F*^2^) = 0.147	H atoms treated by a mixture of independent and constrained refinement
*S* = 1.03	*w* = 1/[σ^2^(*F*_o_^2^) + (0.087*P*)^2^] where *P* = (*F*_o_^2^ + 2*F*_c_^2^)/3
1481 reflections	(Δ/σ)_max_ = 0.006
122 parameters	Δρ_max_ = 0.31 e Å^−^^3^
4 restraints	Δρ_min_ = −0.29 e Å^−^^3^

### Special details

**Table:**

Geometry. All s.u.'s (except the s.u. in the dihedral angle between two l.s. planes) are estimated using the full covariance matrix. The cell s.u.'s are taken into account individually in the estimation of s.u.'s in distances, angles and torsion angles; correlations between s.u.'s in cell parameters are only used when they are defined by crystal symmetry. An approximate (isotropic) treatment of cell s.u.'s is used for estimating s.u.'s involving l.s. planes.
Refinement. Refinement of *F*^2^ against ALL reflections. The weighted *R*-factor *wR* and goodness of fit *S* are based on *F*^2^, conventional *R*-factors *R* are based on *F*, with *F* set to zero for negative *F*^2^. The threshold expression of *F*^2^ > 2σ(*F*^2^) is used only for calculating *R*-factors(gt) *etc*. and is not relevant to the choice of reflections for refinement. *R*-factors based on *F*^2^ are statistically about twice as large as those based on *F*, and *R*- factors based on ALL data will be even larger.

### Fractional atomic coordinates and isotropic or equivalent isotropic displacement parameters (Å^2^)

**Table:**

	*x*	*y*	*z*	*U*_iso_*/*U*_eq_	
O1	0.1276 (2)	0.2500	0.91441 (14)	0.0329 (5)	
O2	0.0786 (2)	0.2500	0.36159 (13)	0.0288 (5)	
N1	0.5591 (2)	0.2500	0.83175 (16)	0.0220 (5)	
N2	0.6293 (3)	0.2500	1.06050 (16)	0.0240 (5)	
N3	0.2321 (2)	0.2500	0.74474 (15)	0.0200 (5)	
H3N	0.327 (2)	0.2500	0.7099 (19)	0.024*	
N4	0.0712 (2)	0.2500	0.68916 (15)	0.0193 (4)	
C1	0.4324 (3)	0.2500	0.90152 (18)	0.0185 (5)	
C2	0.7208 (3)	0.2500	0.87780 (19)	0.0227 (5)	
H2	0.8148	0.2500	0.8320	0.027*	
C3	0.7549 (3)	0.2500	0.99078 (19)	0.0235 (5)	
H3	0.8717	0.2500	1.0195	0.028*	
C4	0.4676 (3)	0.2500	1.01421 (18)	0.0206 (5)	
H4	0.3738	0.2500	1.0602	0.025*	
C5	0.2483 (3)	0.2500	0.85506 (18)	0.0199 (5)	
C6	0.0729 (3)	0.2500	0.58476 (18)	0.0210 (5)	
H6	0.1803	0.2500	0.5519	0.025*	
C7	−0.0876 (3)	0.2500	0.51541 (19)	0.0203 (5)	
C8	−0.0819 (3)	0.2500	0.40041 (19)	0.0214 (5)	
C9	−0.2344 (3)	0.2500	0.3333 (2)	0.0265 (6)	
H9	−0.2304	0.2500	0.2560	0.032*	
C10	−0.3910 (3)	0.2500	0.3800 (2)	0.0362 (7)	
H10	−0.4949	0.2500	0.3343	0.043*	
C11	−0.3992 (3)	0.2500	0.4929 (2)	0.0440 (8)	
H11	−0.5078	0.2500	0.5243	0.053*	
C12	−0.2482 (3)	0.2500	0.5589 (2)	0.0325 (6)	
H12	−0.2542	0.2500	0.6361	0.039*	
C13	0.088612 (1)	0.2500	0.244660 (1)	0.0341 (6)	
H13A	0.2108	0.2500	0.2292	0.051*	
H13B	0.0328	0.3754	0.2135	0.051*	

### Atomic displacement parameters (Å^2^)

**Table:**

	*U*^11^	*U*^22^	*U*^33^	*U*^12^	*U*^13^	*U*^23^
O1	0.0181 (9)	0.0612 (11)	0.0196 (9)	0.000	0.0021 (7)	0.000
O2	0.0280 (10)	0.0415 (10)	0.0168 (9)	0.000	0.0011 (7)	0.000
N1	0.0167 (10)	0.0290 (10)	0.0200 (10)	0.000	−0.0011 (8)	0.000
N2	0.0238 (11)	0.0287 (10)	0.0190 (11)	0.000	−0.0020 (8)	0.000
N3	0.0144 (10)	0.0297 (10)	0.0154 (10)	0.000	−0.0028 (7)	0.000
N4	0.0164 (10)	0.0217 (9)	0.0191 (10)	0.000	−0.0041 (8)	0.000
C1	0.0175 (12)	0.0199 (11)	0.0180 (12)	0.000	0.0003 (9)	0.000
C2	0.0170 (11)	0.0301 (12)	0.0207 (12)	0.000	−0.0017 (9)	0.000
C3	0.0166 (12)	0.0312 (12)	0.0221 (13)	0.000	−0.0039 (10)	0.000
C4	0.0192 (12)	0.0261 (11)	0.0164 (11)	0.000	0.0002 (9)	0.000
C5	0.0176 (12)	0.0234 (11)	0.0184 (12)	0.000	−0.0004 (9)	0.000
C6	0.0205 (12)	0.0227 (11)	0.0194 (12)	0.000	−0.0008 (9)	0.000
C7	0.0218 (12)	0.0200 (11)	0.0182 (11)	0.000	−0.0049 (9)	0.000
C8	0.0257 (13)	0.0188 (11)	0.0192 (12)	0.000	−0.0015 (9)	0.000
C9	0.0339 (14)	0.0244 (12)	0.0195 (12)	0.000	−0.0102 (11)	0.000
C10	0.0234 (13)	0.0512 (16)	0.0317 (15)	0.000	−0.0145 (11)	0.000
C11	0.0200 (14)	0.079 (2)	0.0323 (16)	0.000	−0.0017 (12)	0.000
C12	0.0262 (14)	0.0484 (15)	0.0227 (13)	0.000	−0.0003 (11)	0.000
C13	0.0406 (16)	0.0459 (15)	0.0160 (13)	0.000	0.0030 (11)	0.000

### Geometric parameters (Å, °)

**Table:**

O1—C5	1.224 (3)	C4—H4	0.9500
O2—C8	1.363 (3)	C6—C7	1.459 (3)
O2—C13	1.4395 (16)	C6—H6	0.9500
N1—C2	1.341 (3)	C7—C12	1.388 (3)
N1—C1	1.346 (3)	C7—C8	1.412 (3)
N2—C3	1.339 (3)	C8—C9	1.394 (3)
N2—C4	1.342 (3)	C9—C10	1.378 (4)
N3—C5	1.348 (3)	C9—H9	0.9500
N3—N4	1.381 (2)	C10—C11	1.388 (4)
N3—H3N	0.875 (10)	C10—H10	0.9500
N4—C6	1.280 (3)	C11—C12	1.377 (4)
C1—C4	1.389 (3)	C11—H11	0.9500
C1—C5	1.502 (3)	C12—H12	0.9500
C2—C3	1.391 (3)	C13—H13A	0.9800
C2—H2	0.9500	C13—H13B	0.9800
C3—H3	0.9500		
			
C8—O2—C13	117.35 (16)	N4—C6—H6	119.5
C2—N1—C1	115.86 (19)	C7—C6—H6	119.5
C3—N2—C4	115.54 (19)	C12—C7—C8	118.2 (2)
C5—N3—N4	120.89 (17)	C12—C7—C6	122.0 (2)
C5—N3—H3N	117.6 (17)	C8—C7—C6	119.8 (2)
N4—N3—H3N	121.5 (17)	O2—C8—C9	123.6 (2)
C6—N4—N3	114.97 (17)	O2—C8—C7	116.0 (2)
N1—C1—C4	121.9 (2)	C9—C8—C7	120.3 (2)
N1—C1—C5	118.47 (19)	C10—C9—C8	119.5 (2)
C4—C1—C5	119.63 (19)	C10—C9—H9	120.2
N1—C2—C3	121.9 (2)	C8—C9—H9	120.2
N1—C2—H2	119.1	C9—C10—C11	121.0 (2)
C3—C2—H2	119.1	C9—C10—H10	119.5
N2—C3—C2	122.5 (2)	C11—C10—H10	119.5
N2—C3—H3	118.8	C12—C11—C10	119.3 (2)
C2—C3—H3	118.8	C12—C11—H11	120.4
N2—C4—C1	122.4 (2)	C10—C11—H11	120.4
N2—C4—H4	118.8	C11—C12—C7	121.7 (2)
C1—C4—H4	118.8	C11—C12—H12	119.1
O1—C5—N3	124.9 (2)	C7—C12—H12	119.1
O1—C5—C1	121.5 (2)	O2—C13—H13A	108.1
N3—C5—C1	113.65 (18)	O2—C13—H13B	109.6
N4—C6—C7	121.02 (19)	H13A—C13—H13B	109.4
			
C5—N3—N4—C6	180.0	N4—C6—C7—C12	0.0
C2—N1—C1—C4	0.000 (1)	N4—C6—C7—C8	180.0
C2—N1—C1—C5	180.0	C13—O2—C8—C9	0.0
C1—N1—C2—C3	0.000 (1)	C13—O2—C8—C7	180.0
C4—N2—C3—C2	0.000 (1)	C12—C7—C8—O2	180.0
N1—C2—C3—N2	0.000 (1)	C6—C7—C8—O2	0.0
C3—N2—C4—C1	0.000 (1)	C12—C7—C8—C9	0.0
N1—C1—C4—N2	0.000 (1)	C6—C7—C8—C9	180.0
C5—C1—C4—N2	180.0	O2—C8—C9—C10	180.0
N4—N3—C5—O1	0.0	C7—C8—C9—C10	0.0
N4—N3—C5—C1	180.0	C8—C9—C10—C11	0.0
N1—C1—C5—O1	180.0	C9—C10—C11—C12	0.0
C4—C1—C5—O1	0.0	C10—C11—C12—C7	0.0
N1—C1—C5—N3	0.0	C8—C7—C12—C11	0.0
C4—C1—C5—N3	180.0	C6—C7—C12—C11	180.0
N3—N4—C6—C7	180.0		

### Hydrogen-bond geometry (Å, °)

**Table:**

*D*—H···*A*	*D*—H	H···*A*	*D*···*A*	*D*—H···*A*
N3—H3n···N1	0.882 (18)	2.27 (2)	2.685 (3)	108.9 (19)
C2—H2···O1^i^	0.95	2.57	3.160 (3)	121
C3—H3···O1^i^	0.95	2.44	3.103 (3)	127
C9—H9···N2^ii^	0.95	2.56	3.437 (3)	153

Symmetry codes: (i) *x*+1, *y*, *z*; (ii) *x*−1, *y*, *z*−1.
